# Quantifying Slowness in Parkinson Disease Using a Serious Game: Cross-Sectional Study

**DOI:** 10.2196/79463

**Published:** 2026-02-25

**Authors:** Luanne Cardoso Mendes, Ariana Moura Cabral, Camille Marques Alves, Isabela Alves Marques, Alberto López Delis, Yann Morère, Adriano de Oliveira Andrade

**Affiliations:** 1 Centre for Innovation and Technology Assessment in Health, Faculty of Electrical Engineering, Universidade Federal de Uberlândia, 2121 João Naves de Ávila Avenue, Santa Mônica, Uberlândia, 38408-100, Brazil, +55 34 3239 4729; 2 Medical Biophysics Center, Universidad de Oriente, Santiago de Cuba, Cuba; 3 Laboratoire de Conception, d’Optimisation et de Modélisation des Systèmes, Université de Lorraine, Metz, France

**Keywords:** Parkinson disease, bradykinesia, objective evaluation, serious game, slowness

## Abstract

**Background:**

Slowness in voluntary movements is a hallmark of Parkinson disease (PD); yet, objective measurement outside clinical settings is limited. Serious games represent a promising alternative to extract motor performance metrics during interactions. However, evidence on the effectiveness of these games in discriminating motor performance between individuals with and those without PD is still scarce.

**Objective:**

This study aimed to objectively assess slowness using the RehaBEElitation serious game, based on in-game estimated measurements.

**Methods:**

The study included 15 individuals with mild to moderate PD (Hoehn and Yahr I-III), assessed in both ON and OFF medication states, and 15 age- and gender-matched healthy controls (10 men and 5 women; mean 66.27, SD 9.13 years; range 45-82 years). All participants played each phase of the game on the easiest level. Slowness was evaluated by detecting the voluntary movement of the gyroscope signals using the singular spectrum analysis method. The response time (RT) and angular velocity (AV) of the participants while playing RehaBEElitation were estimated. Group-level comparisons were performed to investigate the presence of slowness patterns across conditions. The Kruskal-Wallis test and Wilcoxon signed rank test with Bonferroni correction were used to confirm the differences between groups, and the effect size was estimated using eta square (η^2^). Spearman correlation analyses were conducted to examine associations between the RT and AV and the Movement Disorder Society-Unified Parkinson’s Disease Rating Scale (MDS-UPDRS) motor items.

**Results:**

Groups were age-homogeneous (*P*>.05). Participants with PD had significantly higher scores on MDS-UPDRS Part III in the OFF state compared to the ON state of medication (mean difference 2.86, 95% CI 2-12; *P*<.05). RT was generally shorter and AV higher in controls than in participants with PD. In the PD group, RT decreased and AV increased from OFF to ON states, reflecting an improvement in motor performance. Significant differences in RT were observed between groups in all phases of the game, with effect sizes ranging from small to moderate (η^2^=0.0239‐0.0650). AV differed markedly between groups in phase 4, with a large effect size (η^2^=0.404). Correlation analyses revealed weak positive associations between RT and MDS-UPDRS items, while AV showed strong negative correlations with each motor item and the summary score for bradykinesia.

**Conclusions:**

This study proposes a new method to assess slowness in PD by using inertial sensor data to extract objective motor measures (RT and AV) during a serious game, allowing continuous and quantitative evaluation beyond traditional clinical scales and tests. The findings demonstrate that RT and AV extracted from gameplay can detect slowness-related motor differences, supporting the RehaBEElitation serious game as an alternative and objective digital biomarker, with potential applications in both clinical and home-based monitoring of symptom progression.

## Introduction

Serious game–based interventions have attracted considerable attention from the medical community. These technologies are defined as digital games developed with a primary purpose beyond entertainment, typically aimed at supporting education, training, or promoting behavioral change. In addition to providing rewarding experiences, these technologies can encourage interaction and enjoyable gameplay [1]. People with a variety of illnesses, including Parkinson disease (PD), can benefit from exercise training, symptom monitoring, and health improvement through serious games’ engaging gaming dynamics [2]. The loss of dopaminergic neurons in the mesencephalon’s substantia nigra is a hallmark of PD [3]. The lack of dopamine hinders the ability to execute and control movements, causing a number of identifiable motor symptoms, including bradykinesia, tremor, and rigidity [4].

The primary motor deficit seen in people with PD is bradykinesia, which is the incapacity to prepare, start, and carry out motions [5]. More specifically, bradykinesia can be understood as a failure of the basal ganglia to reinforce the cortical mechanisms that prepare and execute commands to move [6]. In this context, slowness in performing voluntary movements, prolonged reaction time (defined as the time between the stimulus presentation and the start of the movement), prolonged movement time (defined as the time between the start and finish of the movement), and, ultimately, prolonged response time (RT), which is the sum of reaction time and movement time, are the main characteristics of bradykinesia [7]. RT represents the capacity of an individual to identify pertinent information sources, interpret that information, and apply it to produce a suitable movement response [8]. Since PD impairs both reaction and movement time [7], RT could be an accurate indicator of slowness severity.

The most popular and well-accepted technique for the clinical evaluation of bradykinesia is the Movement Disorder Society-Unified Parkinson’s Disease Rating Scale (MDS-UPDRS) [9]. The capacity of the patient to make quick, repeated, and alternating movements—like tapping fingers, opening and closing hands, and pronating and supinating the forearms—is the base for the clinical assessment of this symptom. To determine the severity of the condition, the clinician grades these movements from 0=normal to 4=severe [10]. However, this kind of evaluation is subjective and inaccurate due to a number of factors, including the evaluator’s expertise level, patient compliance, and individual bias [11]. Furthermore, the clinical evaluation is unable to precisely characterize the severity of a symptom in a patient [12].

Inertial sensors can address the limitations of qualitative visual scales by providing objective motion measurements [12]. Recent studies confirm that inertial measurement units (IMUs) accurately quantify bradykinesia in PD in standardized tasks and remote monitoring. In upper limbs, studies such as Bremm et al [13] confirm that the extraction of metrics of speed, amplitude, and rhythm in tasks such as finger tapping, pronation-supination, and opening or closing the hand showed consistent correlations with clinical scales (such as the MDS-UPDRS). In terms of mobility and overall function, Hong et al [14] used IMUs to capture walking speed, cadence, and stride length, as well as sit-to-stand performance, suggesting that wearable sensors provide a comprehensive and objective approach to assessing a wide range of motor symptoms in PD.

Recent studies in the field of continuous monitoring have highlighted the potential of wearable devices and smartphones to remotely detect patterns of slow movement and reduced range of motion throughout the day. The results demonstrated the feasibility of more accurate and personalized monitoring, reducing the need for frequent in-person visits and supporting therapeutic decisions [15].

Despite covering different tasks and contexts, and demonstrating that the use of devices with IMUs is a promising alternative that can identify and quantify slowness, no study has been conducted to evaluate this symptom with data collected from people with PD during interaction with serious games. In other words, IMU-based strategies are already valid and sensitive for assessing bradykinesia, but they have not yet been applied in gamified protocols, highlighting a methodological gap and a clear opportunity for innovation. Objective evaluation supplements the clinical evaluation [16].

This shortcoming motivated us to develop a serious game, so-called RehaBEElitation (Institute of Industrial Property—INPI registration: BR512021001975-0), which features 4 phases, to assess bradykinesia objectively and collaboratively [17]. The system uses a custom wearable glove interface with conductive threads and inertial sensors. According to the evaluation of the game’s usability, RehaBEElitation is a serious game with an easy-to-understand narrative that meets adequately with the players’ mental models, which aligns with the players’ expectations of movement-control correspondence (eg, hand extension and flexion to move the bee up and down, adduction and abduction to move it left and right, and a pinch gesture to collect nectar—represented in the game as a droplet). This facilitated the interaction of individuals with the serious game and helped the system as a whole gain widespread acceptance [17,18].

This study extends a previous work [19] by evaluating bradykinesia across all 4 game phases (rather than just 2) using a time-based variable and introducing an improved human-machine interface (HMI) device. Additionally, because the signals captured by the HMI device consist of both the voluntary movement the person makes to play the game and the involuntary movement caused by the disease (ie, tremor), it is necessary to separate the involuntary movement from the detected signals since it could affect the slowness estimation. Another distinction in this work is the detailed explanation of the signal processing strategy used to exclude tremors from the collected data and, additionally, the inclusion of a correlation analysis between slowness-related variables and the clinical scale.

A key advantage of the RehaBEElitation serious game is the recording of real-time motion time series during interaction, enabling more in-depth and accurate analysis of the collected data. A recent literature review conducted by our group [20] revealed that previous studies evaluated PD symptoms or monitored health conditions related to the disease using serious games combined with physical instruments, such as the Box and Blocks Test [21], the Purdue Pegboard Test [22], the Nine Hole Peg Test [23], the Time Up and Go test [24], the Maximum Step Length test [25], or the Trail Making Test [26]. None of these studies used data concerning the neuromuscular response of people with PD for the assessments. In the study, Sánchez-Herrera-Baeza et al [22], for example, players performed a test that assesses manual dexterity (the Box and Blocks Test) before and after interactions with the serious game developed, and the authors evaluated manual dexterity by comparing the results of the test performed at these different moments.

Similarly, Jäggi et al [27] conducted a pilot study with hospitalized patients with typical and atypical forms of PD using cognitive and motor exergames. Several cognitive tests (eg, Go/No-Go test, Reaction Time test, and Trail Making Test) and motor tests (eg, Preferred Gait Speed, Maximum Gait Speed, and Time Up and Go) were performed before and after playing the game to assess the motor and cognitive characteristics of the participants. Bégel et al [28] tested the effect of fine motor skills training, administered through serious games played on tablets, on the walking abilities of people with PD. Participants underwent motor performance tests before and after training with 2 games. The authors assessed the participants’ gait and mobility using the Battery for the Assessment of Auditory Sensorimotor and Timing Abilities and the Beat Alignment Test, respectively. Finally, Ganzeboom et al [29] applied a serious game aimed at speech training in patients with PD-associated dysarthria. Each participant recorded 24 speech utterances before and after playing the game. The participants’ speech was then evaluated for intelligibility by untrained listeners, comparing it with corresponding utterances made by a healthy speaker.

In contrast, the system developed in this study captures inertial sensor data that reflect neuromuscular activity to control the game avatar, enabling the objective quantification of motor symptoms, such as bradykinesia, through the extraction and analysis of variables that characterize the symptom. This distinctive capability constitutes a key differentiator of the present research. Accordingly, the purpose of this study was to objectively assess slowness in people with PD through their interaction with the RehaBEElitation serious game, using neuromuscular data to provide a precise and engaging way to quantify motor symptoms. Importantly, our intention is not to replace the clinical evaluation provided by the MDS-UPDRS, but rather to complement it by adding a fine-grained, performance-based measure obtained directly from the participants’ motor behavior during the game.

## Methods

This is an observational and cross-sectional study focused on the objective assessment of slowness, one of the main characteristics of PD. The experimental design involves collecting data from healthy individuals and individuals with PD in 2 different conditions: before taking medication that controls the motor symptoms of the disease (OFF state) and after administering this medication (ON state).

### Ethical Considerations

This research was approved by the Ethics Committee for Human Research at the Federal University of Uberlandia (UFU; protocol: 43229921.8.0000.5152). All participants provided written informed consent prior to enrollment in the study. The informed consent included authorization for the use of anonymized data for research and publication purposes. The experimental protocol was conducted in accordance with national and international ethical guidelines for human research, such as the Declaration of Helsinki. All collected data were deidentified prior to analysis. No information that could directly or indirectly identify participants was stored or included in the manuscript. Data were handled in secure and access-controlled institutional repositories, following best practices for confidentiality protection. In addition, participants did not receive any monetary compensation for participation.

### Serious Game and Interface Device

RehaBEElitation is a bee-themed serious game designed to rehabilitate and monitor people with PD. The bees represent diligence, commitment, and hard work, qualities that people with PD really value during their recovery. The game was developed by a multidisciplinary team using Unity 3D. The player has to control the movements of a bee in a 3D environment. The game tasks were designed to require users to perform the identical motions as those found in the reference tool used to evaluate individuals with PD (MDS-UPDRS Part III): hand opening and closing, wrist extension and flexion, wrist adduction and abduction, finger tapping, and forearm supination and pronation. RehaBEElitation presents 4 phases (Multimedia Appendix 1), and each one symbolizes a real-world bee worker’s task [17]. The objectives of each phase are as follows:

- Phase 1: pollinating the flowers—The objective is to collect pollen from a flower and deposit it in another flower. To move the bee up and down, the player must perform the movements of wrist extension and flexion; and to move the bee to the left and right, the movements of wrist adduction and abduction. The player must move the bee to a flower containing pollen (indicated by yellow arcs) and close the hand to catch it. Then, with the hand closed, the player must move the bee to a flower that does not have pollen (with green arcs) and open the hand to deposit it.
- Phase 2: feeding the larvae—The objective is to feed the larvae. The bee moves around the scene (up, down, left, and right) only if the player’s hand is closed. The player must place the bee in front of a larva and open and close his or her hand to feed the larva.
- Phase 3: collecting the nectar—The objective is to collect nectar from flowers. Flowers that have nectar are indicated by drops of water. The player must guide the bee to a flower that has nectar and perform the finger-tapping movement to collect it.
- Phase 4: drying the nectar—The objective is to dry the nectar to produce honey. The player must go to honeycombs containing nectar (indicated by a luminous reflection) and perform forearm supination and pronation movement (as a single movement) to make the bee flap its wings faster and dry the nectar.

An HMI was created to promote communication between the movements of the real environment and the game (Multimedia Appendix 2). It consists of a glove with inertial sensors, housed in a small case attached to the glove on the back of the hand, capable of estimating hand orientation; and conductive thread sewn on the glove, toward the palm of the hand and the fingers, capable of estimating finger tapping movements and hand opening and closing.

### Experimental Protocol

#### Overview

The experimental group (EG) in this study consisted of 15 participants with PD, while the control group (CG) consisted of 15 healthy participants. Age and gender pairs were formed among the group volunteers (10 men and 5 women; mean 66.27, SD 9.13 years; range 45‐82 years). The eligibility criteria for participants were as follows: a clinically confirmed diagnosis of PD, be aged between 40 and 100 years of age, regular use of PD medication (typically levodopa and/or dopamine agonists), have a mild to moderate PD stage (Hoehn and Yahr levels I, II, and III) [30], score higher than 19 on the Mini Mental State Examination [31], not have severe hearing or vision impairments (hearing acuity was assessed using the Self-Declaration Questionnaire on Hearing Loss, and visual acuity was assessed using the Snellen Optometric Scale), and not have another neurological disease history.

A convenience sampling strategy was adopted due to the specific eligibility criteria and availability of participants in the recruitment context. The sample size was defined based on previous studies with similar experimental designs that investigated motor performance and bradykinesia or slowness of the upper limbs in PD, and which demonstrated adequate power to detect differences between groups in movement-related variables [22,27,28]. The survey was disseminated at care centers for people with PD and through the communication channels of the UFU. Those interested in participating contacted the research team directly to schedule an appointment.

Data collection was conducted in a controlled environment at the Centre for Innovation and Technological Assessment in Health at UFU between February and March 2022. Upon arrival at the site, participants were welcomed by the research team, who presented the study objectives and provided necessary clarifications before signing the Informed Consent Form. Only after formal agreement to participate and verification of the inclusion criteria did the experimental procedures and data collection begin.

The participants were instructed to sit on a chair a meter from the screen that displayed the serious game. The limb most affected by the disease, as identified by a clinical assessment, was then fitted with the HMI device. To isolate the necessary movements and prevent compensating with another body part during movement execution, the limb was placed on a forearm support (Multimedia Appendix 3).

The participants were asked to play each phase of the serious game for 5 minutes or until they fulfilled their objectives. Data collection was performed in the ON and OFF states of medication for the EG. The first session was performed with individuals in the OFF state, after arriving at the collection site, in the morning. The ON state occurs when disease symptoms are under medication control, and the OFF state occurs when symptoms are not properly controlled by medication [16]. The EG volunteers were instructed to arrive in the morning having abstained from their PD medication for at least 8 hours, ensuring the re-emergence of motor symptoms and standardizing the OFF condition. After completing the first session in the OFF state, the EG participants took their usual medication (levodopa-based, such as Prolopa BD or other Prolopa presentations), and approximately 45 minutes later (once the therapeutic effects began to emerge), they performed the second session in the ON state, when symptoms were expected to be adequately controlled by medication.

The MDS-UPDRS Part III [9] was applied to the EG by an independent physiotherapist, who was not involved in the development or execution of the intervention protocols. Assessments were conducted in each session, before starting the game, that is, in both the ON and OFF states of the medication. The MDS-UPDRS was not applied to the CG. The CG participants interacted with the RehaBEElitation serious game in a single session using their dominant upper limb. All participants played the game at the easiest difficulty level.

#### Slowness Evaluation

First, bradykinesia was clinically assessed using the scores related to items 3.4a to 3.6b of the MDS-UPDRS Part III for the EG in both the ON and OFF states of medication. These items assess: 3.4a and 3.4b—tapping the fingers of the right and left hands, respectively; 3.5a and 3.5b—movements of the right and left hands, respectively; and 3.6a and 3.6b—pronation-supination movements of the right and left hands, respectively. The average scores of these items were considered for each medication state. In addition, slowness was objectively assessed for all participants of the study in 2 ways: by calculating the RT and by estimating the individuals’ angular velocity (AV) of movement. Methodological details on how each variable was estimated are provided in the subsequent sections.

As described previously, RT involves both reaction and movement time, since RT is equal to reaction time added to movement time (Multimedia Appendix 4). Across all phases, when the participant correctly positioned the avatar on the target, a stimulus was presented to indicate the precise moment for movement execution. Accordingly, participants learned to align the avatar with the designated location and to initiate the movement only after stimulus onset.

AV (in degrees per second) represents the speed of movement execution [32] (ie, in relation to the RT variable, AV is linked to movement time). For a given movement, the higher the AV, the shorter the time taken to perform the voluntary movement. Thus, if it is verified that the velocity of movement execution was reduced after the use of medication, that is, in the ON state, the time to perform the movement increased, which may suggest a failure in the medication effect.

In addition, RT allows considering aspects related to the player’s reaction time, such as the learning acquired after the first section of interaction with the game. The participant may, for example, decrease his reaction time in the second game session (in the ON state) by learning that, immediately after receiving the visual and sound stimuli, he should perform the movement required to score points in the game in the shortest possible time.

Descriptive and inferential statistical analyses were performed to compare RT and AV between the EG and CG. The normality of data distribution was verified by the Shapiro-Wilk test prior to comparisons between groups, and appropriate parametric or nonparametric tests were adopted according to this result. In this study, the nonparametric Kruskal-Wallis test was used, followed by Wilcoxon signed rank tests with Bonferroni correction for pairwise group comparisons. The effect size was estimated using eta square (η^2^) and interpreted according to Cohen criteria [33], with η^2^ values of approximately 0.01 considered small, around 0.06 considered moderate, and values of ≥0.14 considered large.

Spearman rank-order correlation analyses were conducted to examine associations between the RT and AV variables and the MDS-UPDRS motor items previously described (items 3.4a/b, 3.5a/b, and 3.6a/b), as well as with the summed score of these items (BRADINDEX). For each participant, the median value of the estimated RT and AV data was calculated and used in the correlation analyses. The correlation coefficient (ρ) was classified into 5 categories: very weak (0.00‐0.19), weak (0.20‐0.39), moderate (0.40‐0.59), strong (0.60‐0.79), and very strong (0.80‐1.00) [34]. Signal processing and statistical procedures were performed using the R language for statistical computing (R Foundation for Statistical Computing).

Matching by age and gender during recruitment was used to minimize possible confounding factors, and no subgroup or interaction analyses were conducted due to the exploratory nature of the study and the matching strategy used. There were no missing data, as all attempts were successfully completed and validated during acquisition. Considering that sampling was by convenience, statistical analyses considered independent samples without additional weighting. Sensitivity analyses were not necessary, as all data were maintained and processed consistently throughout the study.

#### RT Estimation

**Figure 1.**
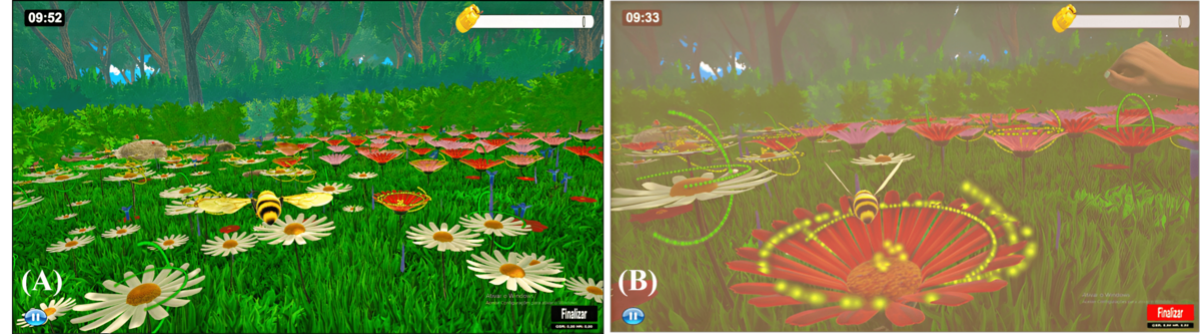
Game screen showing the event to response time estimation. (**A**) Game screen while the player freely controlled the bee in the scenario. In this case, the player has not yet approached the target. (**B**) Game screen when the player reached the target and received the visual and sound stimuli, indicating that he/she could start executing the required movement. At this point, the bee remained static, the screen subtly changed color, a virtual hand appeared in the right corner of the screen, indicating to the player the movement to be performed, and a sound effect was emitted.

RT was measured by defining a binary event in the game that signaled when the player approached the target and executed the movement. This event began when the player was supplied with visual and auditory stimuli, indicating that he or she could perform the movement and ended when the player performed the movement and scored in the game. Figure 1 shows the game screen (during phase 1) at the moment when the event responsible for calculating the RT occurs.

**Figure 2.**
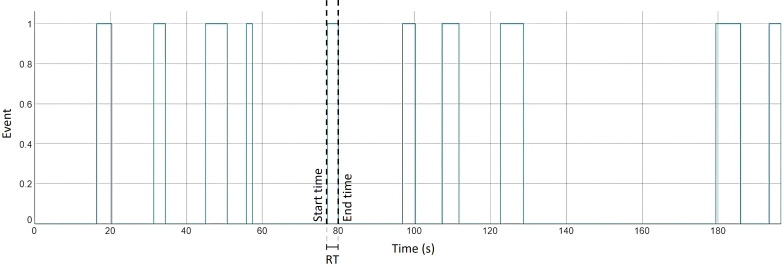
RT estimation from the defined event. Each blue rectangle represents an occurrence of the event, which indicates the duration of the RT. As stated, in this phase, the player hit the target and completed the expected movement 10 times, producing 10 RT values; the fourth occurrence showed the minimum RT, indicating that the player was faster in this instance. RT: response time.

Figure 2 illustrates the occurrence of the described event of a volunteer from the EG in the OFF state during phase 4. This player arrived at the target and executed the movement 10 times (10 occurrences of the binary event). The start and end of each event were identified to determine the start and end times of the event. The duration of each event was then estimated by calculating the difference between the end and start times.

Thus, as RT is the sum of reaction time and movement time, both reaction time and movement time were taken into consideration to estimate the slowness of the individuals. This evaluation was performed for the 4 phases of the game.

#### AV Estimation

##### Overview

Gyroscope signals were used to measure the AV around a reference axis, which indicated how fast the movement was performed [32]. This characteristic was estimated only for phase 4 of the game because it is the only phase that requires the execution of a movement performed around one of the measurement axes of the inertial sensor (forearm supination and pronation) to score in the game. In Multimedia Appendix 5, which shows the reference axes of the inertial sensor, it is observed that the supination and pronation movements occur around the Y axis.

As previously described, the motor symptoms of PD can be detected by inertial sensors and recorded as time-series data. These movement signals recorded by the sensors include both voluntary movements of the player to control the bee in the scenario and involuntary movements caused by the disease (such as tremor). The presence of involuntary movements may interfere with the estimation of slowness; hence, they should be disregarded from the collected signals. Thus, to extract only information related to players’ voluntary movements, it was necessary to use a signal processing method capable of performing time series decomposition.

Time series decomposition involves decomposing a time series into several components, each representing one of the underlying oscillatory patterns in a signal. Singular spectrum analysis (SSA) is a decomposition method frequently used in several fields, such as economics, physics, and geography [35]; however, no study applying this method to signals collected from individuals with PD was found in the literature. In view of this gap and the technique’s capacity to robustly decompose complex temporal signals, the SSA method was adopted in this study.

##### Singular Spectrum Analysis

SSA is a nonparametric method for the analysis of time series and digital images. This method decomposes a time series into a set of components that are grouped and interpreted, and the original time series is recovered by summing all its components [35]. SSA can be used to remove all periodic and trend components from a time series, leaving only a particular component, which may have real and interpretable meaning [35]. In this study, decomposition was performed so that slowness could be evaluated objectively by using only the signals of voluntary movement, that is, excluding the influence of tremor.

A general description of the method is presented below [35-37]. SSA performs the 4 steps illustrated in Multimedia Appendix 6. The input X is an ordered collection of N real numbers (eg, a time series or a digital image), and the output is a decomposition of X into a sum of identifiable components: $X=X_{1}+\cdots +X_{m}$.

- Step 1: embedding: The starting point of SSA is the embedding of the time series X into a vector space of dimension L. As an example, $X\;=\;\left(x_{1},...,x_{N}\right)$ and *T*=SSA maps R^N^ to the space of Hankel matrices L×K with equal values on the antidiagonals. Thus, the embedding procedure constructs a sequence of vectors of the original time series using lagged copies of the scalar data. N is the length of the series, L is the length of the window (which is a parameter), and K=N-L+1.

$$T(SSA)(X)=\left(\begin{array}{ccccc} x_{1} & x_{2} & x_{3} & \cdots & x_{k}\\ x_{2} & x_{3} & x_{4} & \cdots & x_{k+1}\\ x_{3} & x_{4} & x_{5} & \cdots & x_{k+2}\\ \vdots & \vdots & \vdots & \ddots & \vdots \\ x_{L} & x_{L+1} & x_{L+2} & \cdots & x_{N} \end{array}\right)$$

- Step 2: decomposition: The result of this step is the decomposition $X=\sum_{i}Xi$,$Xi={\sigma iUiVi}^{T}$, where Ui∈ R^L^ and Vi∈ R^K^ are vectors such that || Ui ||=1 and || Vi||=1 for all i and σi are nonnegative numbers. This step performs the singular value decomposition (SVD) of the trajectory matrix and represents it as a sum of rank-one biorthogonal elementary matrices. Let $S=XX^{T},\;\lambda_{1}\geq \cdots \geq \lambda_{L}\geq 0$, be eigenvalues of the matrix **S**, d=rank $X=\max\{\;j:\lambda_{j}§gt;0\;\}$, U₁,...,Ud be the corresponding eigenvectors, and $V_{j}=X^{⊤}U_{j}\;/\;\sqrt{\lambda_{j}}$, j=1,...,d be factor vectors. Denote $X_{j}=\sqrt{\lambda_{j}}\;U_{j}V_{j}^{T}$. Then, the SVD of the trajectory matrix **X** can be written as $X=X_{1}+\cdots +X_{d}$. The triple ($\sqrt{\lambda_{i}},\;U_{i},V_{i}$) consisting of the singular value $\sigma_{j}=\sqrt{\lambda_{j}}$ , the left singular vector $U_{j}$, and the right singular vector $V_{j}$ of **X** is called the *j*th eigentriple.
- Step 3: grouping: The grouping step corresponds to the division of the elementary matrices $X_{j}$ into several groups and summing the matrices within each group. The grouping procedure partitions the set of indices {1,...,d} into *m* disjoint subsets $\left\{I_{1},\ldots ,I_{m}\right\}$, with each group $I_{k}$ containing a set of principal components $\left\{i_{1},\ldots ,i_{p}\right\}$, representing specific components of the signal. Let $I_{k}=\{i_{1},\ldots ,i_{p}\}$. Then, the resulting matrix $X_{I_{k}}$ corresponding to the group $I_{k}$ is defined as $X_{I_{k}}=X_{i_{1}}+\cdots +X_{i_{p}}$. These matrices are computed for $I_{k}=I_{1},\ldots ,I_{m}$, and the SVD expansion leads to the decomposition $X=X_{I_{1}}+\cdots +X_{I_{m}}$ . Each $X_{I_{k}}$ represents a set of eigentriples, which describes a specific component series in the original time series.

- Step 4: reconstruction: The last step is to reconstruct the components of the original series. This is achieved by diagonal averaging of each $X_{I_{k}}$ to provide the *k*th component of the series X, where the *n*th sample is obtained by averaging over the cross-diagonal i + j = const = n + 1 of $X_{I_{k}}$ . This is because each $X_{I_{k}}$ can be seen as the Hankel matrix for the corresponding embedded component series. Then, the resulting decomposition of the initial object X is $X=X_{1}+\cdots +X_{m}$.

The SSA procedure was applied to identify the tremor-related oscillatory components, which were subsequently removed from the original time series. The resulting residual signal, obtained by subtracting the tremor components from the original data (since the original signal can be reconstructed as the sum of all SSA-decomposed components), represents the tremor-free voluntary movement used for slowness evaluation.

The estimate of SSA was computed in the R language by using the method ssa (from the *Rssa* package), which constructs the ssa object that holds the decomposition and various auxiliary information. The typical call of the ssa function in R is:

s <- ssa(x, L = (N+1) %/% 2, neig=NULL)

The main arguments are (N is the length of the series):

- x is an object to be decomposed (eg, time series).
- L is a window length, which by default is fixed to half the length of the series. However, it is also recommended to use a proportional value to the periodicity of the time series. Very high values of L may mix the trend component with the periodicity components. Therefore, L=250 was used (the sampling frequency of the data).
- neig is the number of eigentriples desired. If neig=NULL, a default value, which depends on L and N, will be used. It was considered neig=NULL.

The function returns an ssa object. The precise layout of the object is hidden, but identifying the indices of the elementary components is necessary for the next step (reconstruction). This identification is performed to find the selection parameters of the principal components to group the elementary time series. The indices of the elementary components can be identified by using the $ operator and by analyzing the plots of the eigenvalues and the correlation matrix of the eigenvalues, namely, s$sigma returns the vector of single values, (s$sigma)^2^ returns the vector of eigenvalues, plot(s) plots the eigenvalues, and plot(wcor(s)) plots the w-correlation matrix.

By definition, components that have similar eigenvalues are used for the grouping of elementary time series that represent the periodic or oscillatory components of the original time series. In other words, eigenvalues with very similar magnitudes are associated with a periodic or sinusoidal component of the signal, in this case, the PD tremor, which represents an involuntary movement.

The reconstruction step is performed with the reconstruct function. The typical call in R is:

r <- reconstruct(s, groups=list(Tremor=c(3:5, 13:16))

The main arguments are as follows:

- s is an ssa object that holds the decomposition.
- groups is a list of numeric vectors consisting of the indices of the elementary components used for the reconstruction; the list entry can be named, and in this example, it was called Tremor. The elementary components with indices 3, 4, and 5 and 13, 14, 15, and 16 (which have similar magnitudes) represent the oscillatory component of the time series, which characterizes the player’s involuntary movement (tremor).

Thus, SSA was applied to the original signal collected during the experiment to extract the tremor. The residual signal after extraction of the involuntary movement represents the voluntary movement used to quantify the slowness of the participants. In other words, the decomposition allowed us to exclude the frequency range associated with tremor while retaining the frequencies representing voluntary motion, which were then used to quantify participants’ slowness. Multimedia Appendix 7 illustrates the decomposition of a gyroscope signal from a participant of the EG. The sum of the voluntary movement and tremor signals results in the original signal.

**Figure 3.**
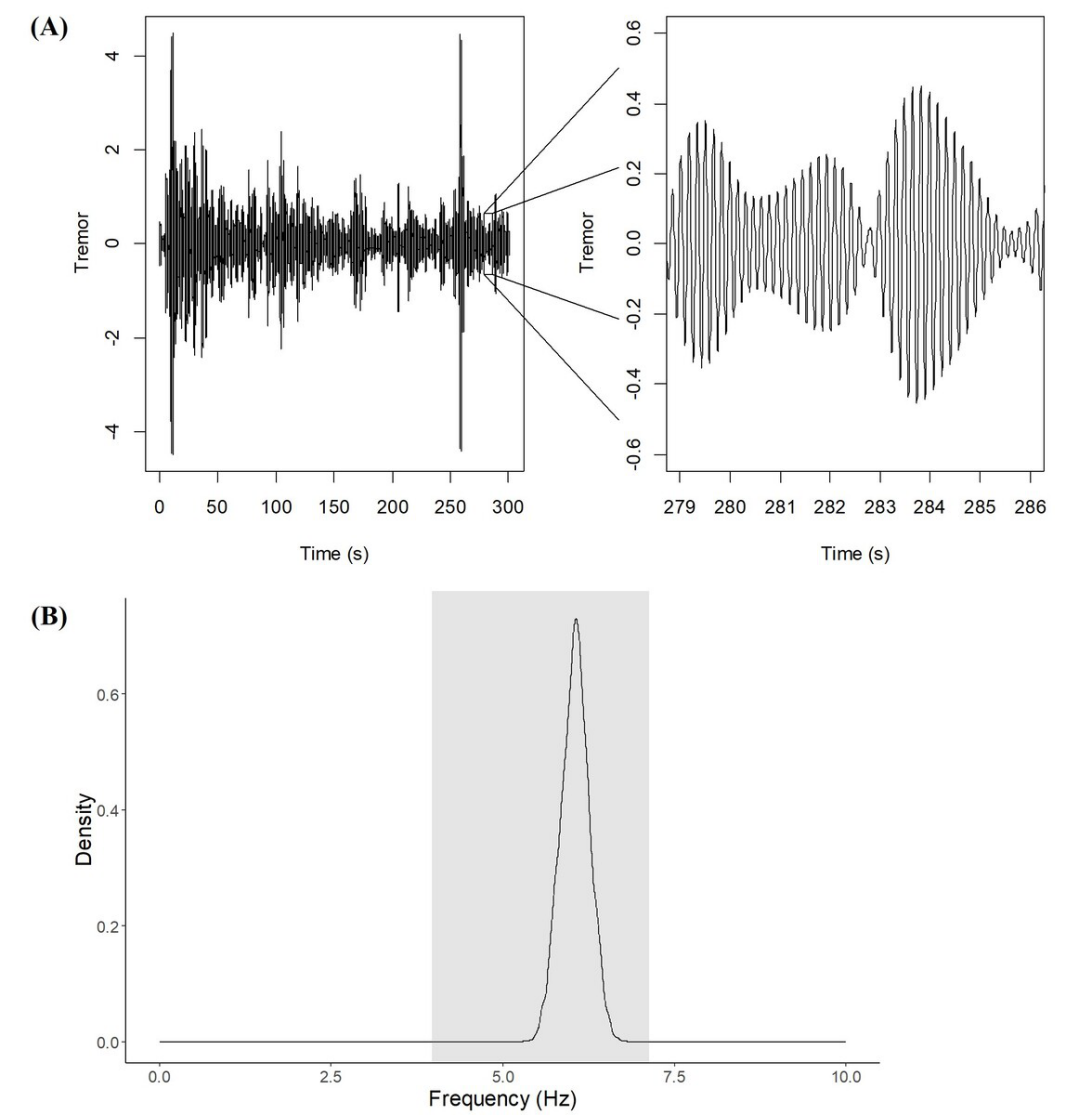
Characteristics of the signal extracted by the singular spectrum analysis decomposition method. (**A**) On the left, the complete tremor signal, and on the right, a zoomed view of the signal. (**B**) Power spectral density of the tremor, confirming that it corresponds to a Parkinsonian tremor, as its dominant frequency lies within the characteristic 4‐7 Hz range.

Figure 3 illustrates a typical tremor signal resulting from the use of SSA. An enlarged view of the tremor signal extracted by the decomposition is shown in Figure 3A (on the right), and the power spectral density of the tremor is depicted in Figure 3B. Figure 3B shows that the density of the tremor extracted by the decomposition ranges between 4 and 7 Hz, which is the frequency range of a tremor originating from PD. This indicates that the method is robust in cleanly separating the oscillatory components of the original signal that truly correspond to PD tremor.

By minimizing the contribution of the higher-frequency components associated with tremor in the spectral profile of the movement, it becomes possible to isolate the signal that truly represents the participant’s voluntary motion. Removing these high-frequency components is essential to ensure that slowness, which is manifested within the voluntary movement itself, can be estimated in a pure and uncompromised manner, without interference from other motor symptoms such as tremor. This allows the control signal used in the game to reflect only the participant’s voluntary movement characteristics, particularly their slowness.

**Figure 4.**
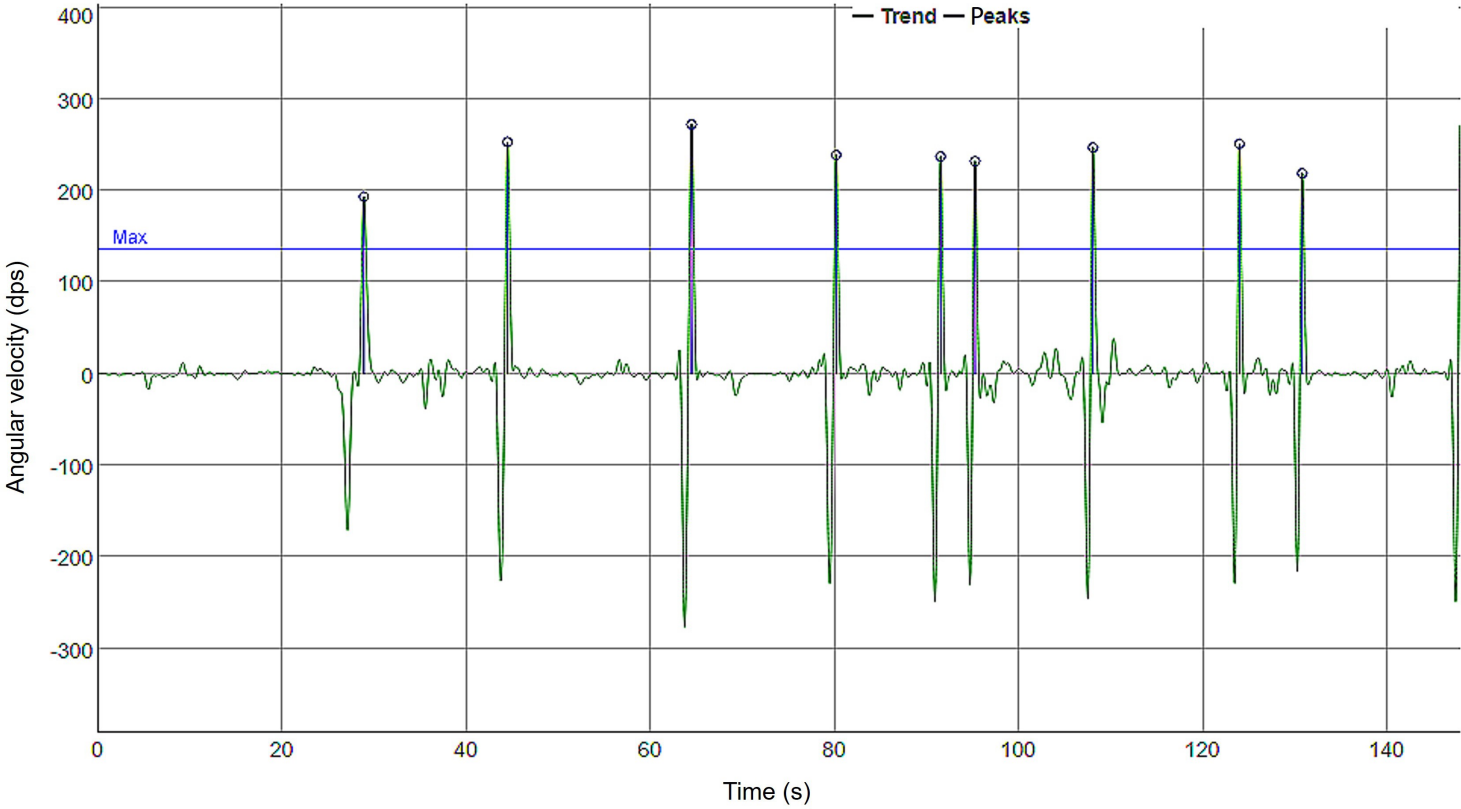
Peak detection in the gyroscope signal, representing the angular velocity of hand movement. Each peak corresponds to a directional change or a complete movement cycle, and higher peak amplitudes indicate faster and more vigorous motion execution.

After applying SSA for signal decomposition, the peaks of the signals representing the voluntary movement were identified and discriminated by using a threshold of 0.5 to capture only the regions of interest and evaluate the participants’ slowness of movement, as depicted in Figure 4. The higher the peak value, the faster the movement was executed.

On the basis of the processes outlined earlier, the influence of involuntary movement arising from PD was mitigated for the assessment of slowness.

To minimize potential sources of bias, certain methodological precautions were implemented. First, standardized protocols were applied during all data collection procedures, including instructions, task execution, and environmental conditions, ensuring consistency among participants. Assessment sessions were conducted individually to avoid external interference and influence from other participants’ performance. Disease severity was controlled by including only individuals classified as having mild to moderate PD (Hoehn and Yahr I-III), thereby reducing heterogeneity in motor impairment. After taking their regular antiparkinsonian medication, the researchers waited for a period of time until each participant subjectively reported improvement in motor symptoms, indicating perception of adequate motor control caused by the medication, minimizing drug-related performance fluctuations. In addition, data processing followed predefined criteria, and the researcher responsible for extracting and organizing the results was unaware of the participants’ identities to reduce observer bias.

## Results

All individuals who expressed interest in participating in the study and were evaluated for eligibility criteria met the established requirements. In total, 15 participants with PD and 15 healthy participants were included and analyzed in the study. Samples from healthy participants were collected only after the participation of an individual with PD of the same age and gender, ensuring pairing between the groups. There were no dropouts or exclusions during the collection process, so all participants fully completed the experimental protocol.

The primary demographic and clinical characteristics of the sample are presented in Table 1. To assess potential differences in age between the PD group and the healthy CG, an ANOVA was conducted, showing no statistically significant difference between the groups (*P*>.05). The Kruskal-Wallis rank-sum test was applied to compare the total MDS-UPDRS Part III scores in the ON and OFF medication states, demonstrating a significant difference between these conditions (*P*<.05). Additionally, an ANOVA performed on the Hoehn and Yahr scores across medication states indicated no significant variation (*P*>.05).

Table 2 displays the slowness assessment results for both the EG and CG. The average RT and AV values for each participant, considering the 4 phases of the game, are presented. The RT values were generally lower for the CG than for the EG, while the AV values were higher for the CG than for the EG in both the ON and OFF states. It is noteworthy that the CG participant with the highest RT also showed the lowest AV (participant 3). However, it is important to emphasize that RT and AV do not have a linear relationship; changes in one measure do not necessarily correspond proportionally to changes in the other, as they capture different aspects of movement performance.

**Table 1.** Main characteristics of study participants, including age, Movement Disorder Society-Unified Parkinson’s Disease Rating Scale (MDS-UPDRS) Part III total score, and Hoehn and Yahr (H&Y) stage.

Control group		Experimental group					
		ON state				OFF state	
ID	Age (years)	ID	Age (years)	MDS-UPDRS Part III	H&Y	MDS-UPDRS Part III	H&Y
1	82	1	57	25	3	61	2
2	64	2	80	25	2	49	2
3	80	3	64	26	2	58	2
4	66	4	77	31	2	44	2
5	51	5	59	24	3	47	2
6	56	6	64	39	2	48	2
7	77	7	70	20	2	41	2
8	62	8	73	42	3	45	3
9	60	9	60	12	2	29	1
10	80	10	66	16	1	29	1
11	69	11	69	37	3	70	2
12	59	12	60	31	2	54	2
13	62	13	50	35	3	59	3
14	55	14	68	38	2	52	2
15	71	15	77	11	1	38	1

**Table 2.** Results of slowness evaluation^a^.

Control group			Experimental group						
			ON state				OFF state		
ID^b^	RT^c^ (seconds)	AV^d^ (degrees per second)	ID	MDS-UPDRS^e^	RT (seconds)	AV (degrees per second)	MDS-UPDRS	RT (seconds)	AV (degrees per second)
1	1.13	308.38	1	3	1.66	305.35	5	3.30	139.49
2	1.30	347.34	2	6	3.73	139.20	9	2.53	177.71
3	3.27	287.36	3	8	2.08	248.31	8	1.63	182.14
4	2.00	400.60	4	6	1.84	351.83	9	1.43	394.17
5	1.59	460.94	5	2	1.98	398.71	3	2.60	306.59
6	1.57	520.40	6	6	1.94	245.04	9	2.42	182.70
7	2.50	391.27	7	4	1.14	234.44	8	2.20	238.86
8	1.95	401.34	8	3	2.15	234.16	6	1.99	301.79
9	1.49	621.23	9	3	1.42	473.47	5	1.93	352.31
10	1.33	506.88	10	2	1.22	272.14	4	1.45	316.26
11	1.70	466.61	11	6	1.26	492.09	9	1.84	327.66
12	1.49	555.35	12	6	2.94	167.36	12	3.70	135.72
13	1.60	565.49	13	6	1.90	152.37	9	1.41	170.01
14	1.49	518.94	14	5	1.90	260.84	9	2.04	252.35
15	1.48	383.09	15	3	1.98	374.44	7	3.51	278.41

a Higher RT and lower AV indicate greater slowness, and higher MDS-UPDRS scores reflect worse motor impairment.

b ID denotes participant identifier.

c RT: response time.

d AV: angular velocity.

e MDS-UPDRS: Movement Disorder Society-Unified Parkinson’s Disease Rating Scale.

Regarding the EG, participants 1, 5, 6, 9, 11, 12, and 15 in the ON state of medication presented lower RT scores and higher AV scores compared to the OFF state. In the OFF state, the individual with the highest degree of disease impairment, according to the clinical evaluation, also presented the largest RT and the smallest AV (participant 12). Participants 3 and 14 increased their AV in the ON state compared to the OFF state but also increased their RT; and participants 2, 7, and 10 decreased their RT in the ON state compared to the OFF state but also decreased their AV. Participants 4, 8, and 13 increased their RT and decreased their AV in the ON state compared to the OFF state.

**Figure 5.**
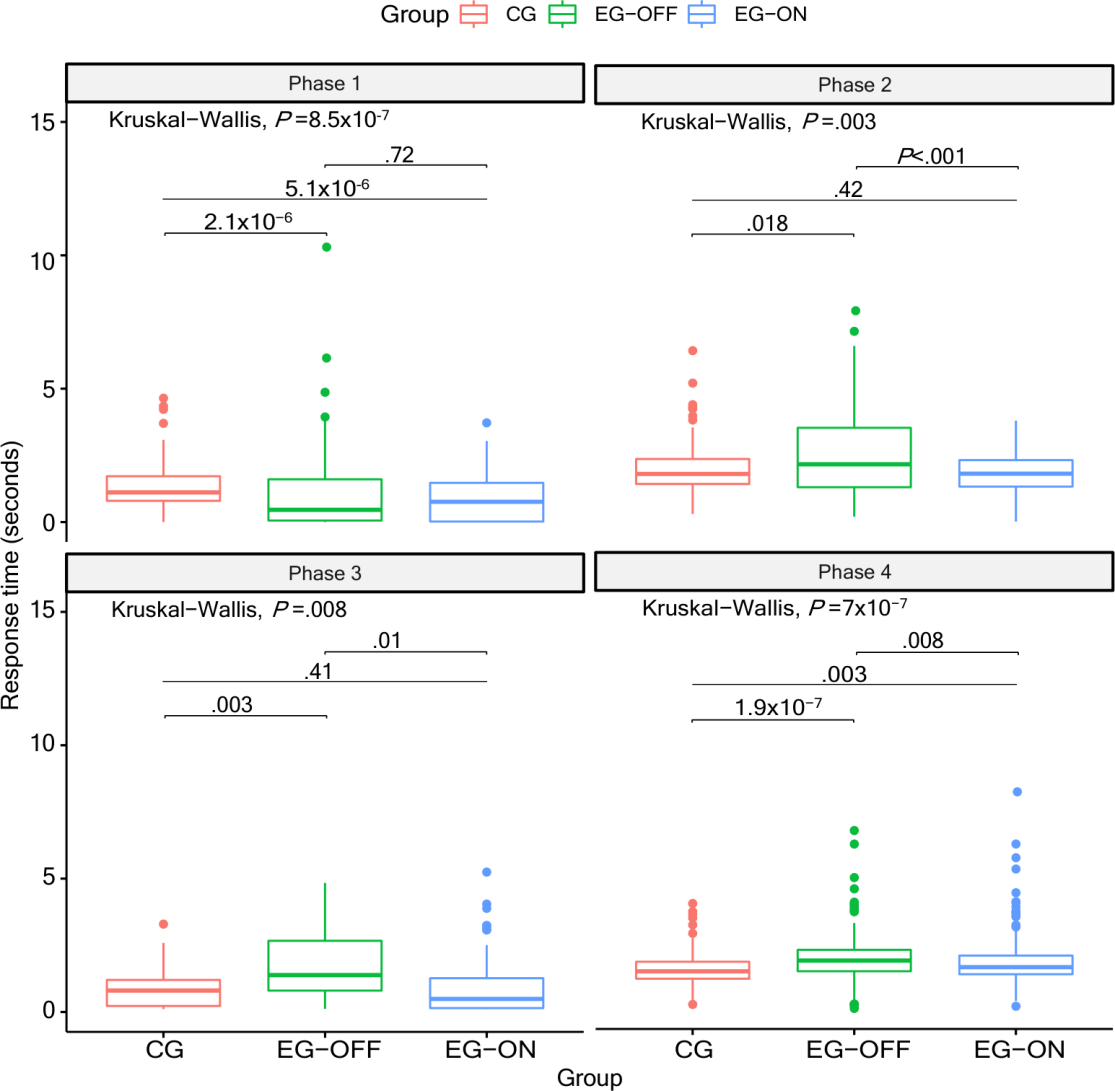
Distribution of estimated response time across participants for each of the 4 game phases, comparing the CG and the EG in the ON and OFF medication conditions. CG: control group; EG: experimental group.

The distribution of RT values for all participants is depicted in Figure 5. Except for phase 1, in all other phases, the CG had the lowest RT, followed by the EG in the ON and OFF states. In phase 1, the EG in the ON state produced the best RT values, followed by the CG and then the EG in the OFF state.

The Kruskal-Wallis test indicated statistically significant differences in RTs among the groups across all phases. Specifically, phase 1 showed *P*=8.5×10^–^⁷ with a small-to-moderate effect size (η^2^=0.0591), and statistically significant differences were found among all groups except between the EG-OFF and EG-ON; phase 2 showed *P*=.0028 with a small effect size (η^2^=0.0239), and no statistically significant differences were observed between the CG and the EG-ON; phase 3 showed *P*=.0081 with a small effect size (η^2^=0.0419), and the comparison between the CG and the EG-ON did not reach statistical significance; and phase 4 showed *P*=7×10^–^⁷ with a moderate effect size (η^2^=0.0650), and statistically significant differences were observed among all groups.

**Figure 6.**
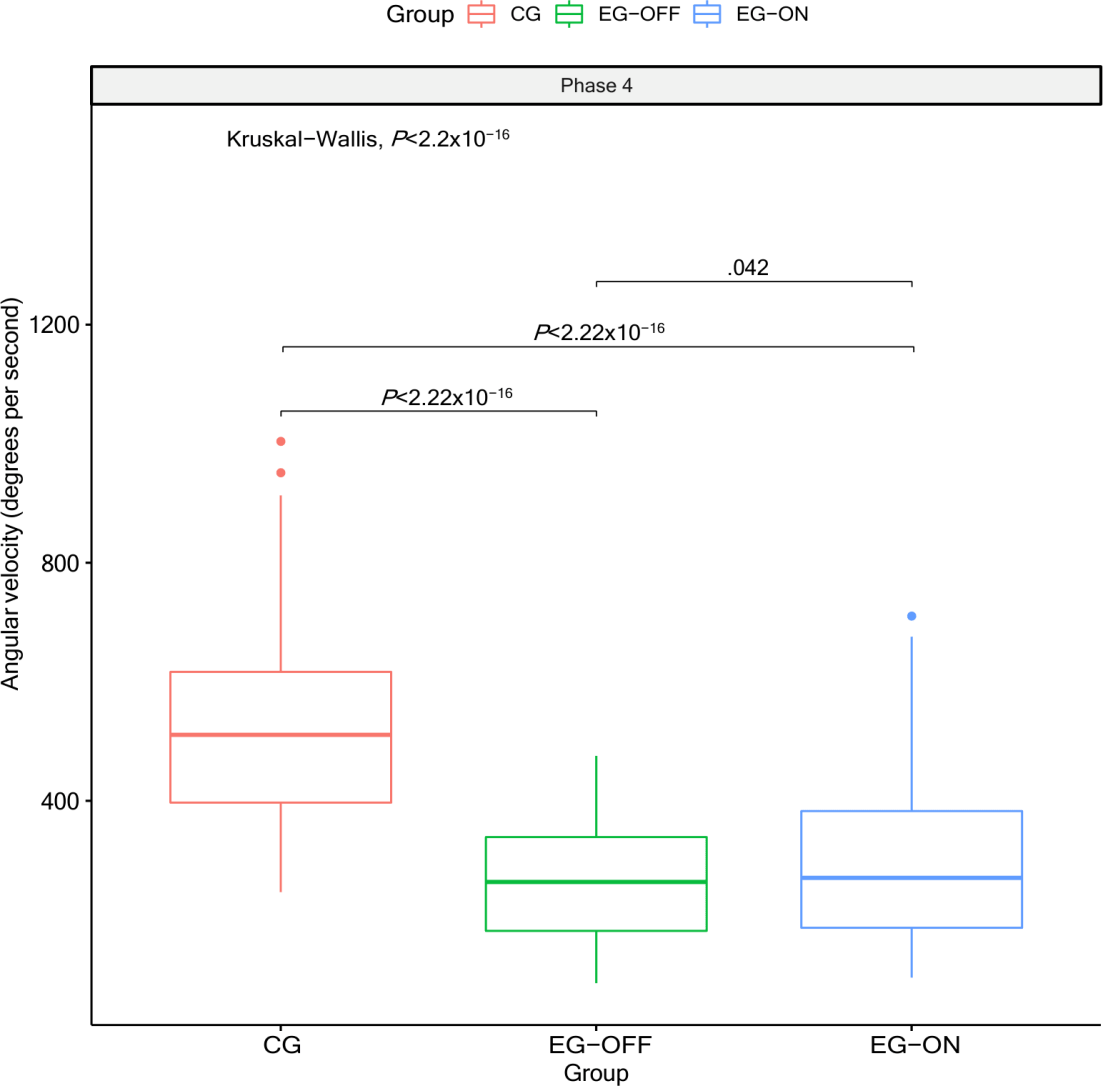
Distribution of angular velocity among participants, comparing the control group and experimental group in both medication states. As noted, angular velocity was estimated exclusively for phase 4.

The distribution of AV data for all participants is shown in Figure 6. Again, the CG had the largest AV, followed by the EG in the ON state, and then by the EG in the OFF state.

A statistically significant difference in AV among the groups was observed using the Kruskal-Wallis test (*P*<2.2×10^–16^), with a large effect size (η^2^=0.404).

It is worth noting that the analyses presented in Figures5  6 were not conducted based on individual trials but rather using aggregate measures per group. Specifically, both figures were constructed based on the median values of each variable within each group, ensuring that differences in the number of trials per participant did not influence the results. Thus, Figure 5 represents the median RT per group in each phase, while Figure 6 presents the median AV per group in phase 4. This procedure ensures a more robust and consistent interpretation of the data, minimizing biases related to intraindividual variability.

**Figure 7.**
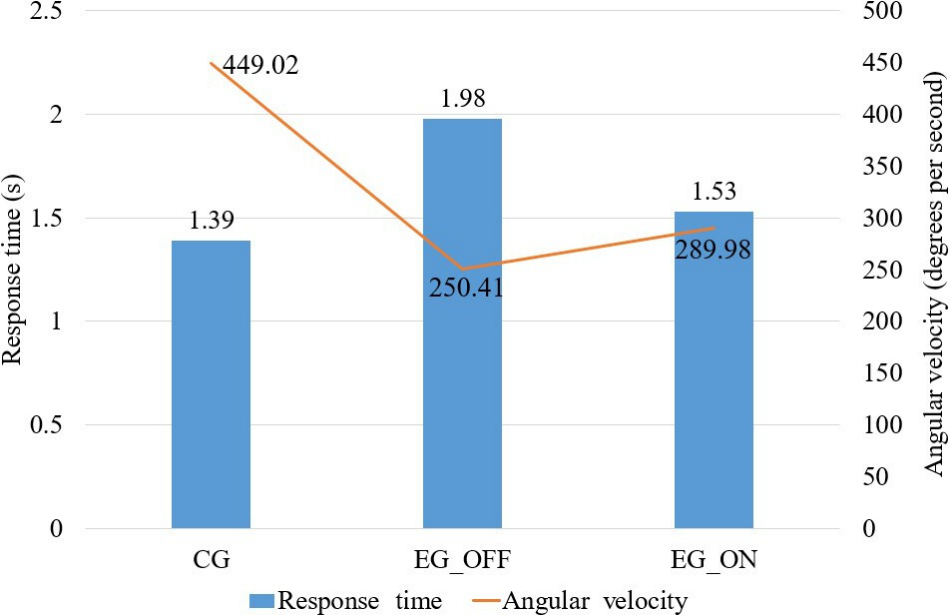
Mean response time and angular velocity values for each group. The CG showed the fastest responses and highest movement speeds, while EG exhibited improved performance in the ON state compared to the OFF state, with lower response times and higher angular velocities. These values provide a quantitative overview of motor performance in all conditions. CG: control group; EG: experimental group.

Figure 7 shows the mean values of RT and AV for all groups. This grouped visualization of the estimated variables (RT and AV) reflects an overall trend in motor performance between the groups. On average, healthy individuals performed faster movements in less time during interaction with the game when compared to participants with PD. In addition, for the EG, the RTs decreased from the OFF state to the ON state, and AVs increased from the OFF state to the ON state. This visualization highlights the overall difference between the groups in terms of RT and AV measurements.

The correlation analysis revealed distinct association patterns between RT and AV and the motor items of the MDS-UPDRS. Table 3 summarizes the corresponding correlation coefficients along with their classifications. RT showed very weak to weak positive correlations with all individual items and with BRADINDEX. In contrast, AV demonstrated strong negative correlations across all MDS-UPDRS items and with the composite bradykinesia index.

**Table 3.** Spearman correlations (ρ) between response time (RT), angular velocity (AV), and the Movement Disorder Society-Unified Parkinson’s Disease Rating Scale (MDS-UPDRS) items.

MDS-UPDRS item	ρ (RT×MDS-UPDRS)	ρ (AV×MDS-UPDRS)
3.4a	0.058	−0.656
3.4b	0.217	−0.617
3.5a	0.114	−0.687
3.5b	0.216	−0.625
3.6a	0.118	−0.725
3.6b	0.215	−0.665
BRADINDEX^a^	0.147	−0.698

a BRADINDEX is the summary of the 3.4a to 3.6b item scores.

## Discussion

On the basis of movement data collected by inertial sensors during participants’ interaction with the serious game, it was possible to measure indicators of slowness in PD. These results demonstrate that metrics extracted from a gamified environment can capture motor changes characteristic of the disease and differentiate individuals with PD from participants in the CG.

### Comparison to Prior Work

Unlike existing literature that relies on subjective scales or external motor tests, RehaBEElitation records inertial signals during gameplay for dual-purpose rehabilitation and monitoring. While the MDS-UPDRS remains the gold standard, its high interexaminer variability and inability to capture daily symptom fluctuations highlight the need for more reliable, objective tools [38,39]. Our system addresses this by capturing direct neuromuscular data through a sensor-integrated glove, ensuring a more precise assessment of impairment.

Despite not addressing the use of serious games, recent studies have demonstrated the use of wearable sensors to assess and monitor the motor signs and symptoms of PD [40,41]. Such technologies can help researchers and health care professionals obtain a more reliable and objective view of disease progression and the severity of motor symptoms. Furthermore, according to Bhidayasiri and Tarsy [16], the successful implementation of wearables to evaluate PD symptoms, in conjunction with the use of established scales such as the MDS-UPDRS, promises to improve the quality of medical care for people with PD. However, despite numerous efforts to create new technologies for bradykinesia assessment, it has not yet been possible to establish one that performs this evaluation objectively, as considerable experience and effort are still required to operate the available technologies [10].

In this study, slowness was assessed using the individuals’ RT, which was calculated by the duration of an event indicating the moment when the player reaches the target and performs the movement, as well as the gyroscope signal collected by the wearable HMI device, which represents the AV of movement. Statistical analysis indicated that the groups of healthy individuals and patients with PD were comparable in terms of age. Overall, as reflected by the MDS-UPDRS III and Hoehn and Yahr scores (Table 1), patients with PD in the OFF medication state exhibited greater impairment than in the ON state.

To obtain a reliable AV estimation, the SSA method was applied to the gyroscope signals to isolate voluntary activity. Several techniques can be used for time series decomposition, such as Fourier analysis, empirical mode decomposition, wavelet transform, kinematic decomposition by submovements, and singular spectrum decomposition (SSD). However, as the recorded signals are nonstationary time series, Fourier analysis is not the ideal option [42]. This approach assumes stationarity, risking the loss of transient information [43]. Moreover, Fourier analysis and empirical mode decomposition can provide data decomposition results with limited physical sense, that is, components with unclear physical meaning. The components retrieved by these techniques can also be affected by mode mixing, again interfering with the physical meaning of individual components, especially in cases where abrupt variations in movement occur [37]. Techniques such as wavelet transform, although suitable for nonstationary data, depend heavily on the prior choice of a mother wavelet, which can introduce bias and hinder the physiological interpretation of the extracted components [40]. The kinematic decomposition by submovements approach is limited by its dependence on ideal models that do not always represent real movements and by its sensitivity to noise during segmentation, which can lead to the misidentification of submovements [44]. Finally, the SSD technique was initially applied to decompose the original signal, but no successful results were obtained. The selection of fundamental parameters in SSD, including window length and elementary component indices, is completely automated, which generated components with no physical meaning.

The SSA method was able to satisfactorily disregard the signal that represents the involuntary movement of the players. The results suggested that this method is particularly useful for this application, as the extracted tremor signal represents a disease-induced tremor (Figure 3). Due to the decomposition, it is possible to separately and independently study the characteristics of the pure movement and of the tremor of individuals with PD by analyzing only the extracted signal of interest.

### Principal Findings

The results indicated in Table 2 showed that participants 1, 5, 6, 9, 11, 12, and 15 in the ON state of medication presented lower RT scores and higher AV scores compared to the OFF state. This result was already expected, since EG participants in the ON state of medication have more reduced motor symptoms than in the OFF state [45]. However, in the ON state, some situations also occurred: participants 2, 7, and 10 reduced RT but also reduced AV, participants 3 and 14 increased AV but also RT, and participants 4, 8, and 13 increased RT and reduced AV. It is known that RT is the sum of reaction time and movement time; AV is more related to movement time.

After taking the medication, participants 2, 7, and 10 had lower RT but performed the movements more slowly; that is, they increased the movement time, which consequently resulted in a decrease in reaction time. Movement time may have increased because the medication is probably not bringing benefits regarding the improvement of motor symptoms in these individuals, which suggests the need to readjust the dosage of the medication or change it for another medication. The reaction time may have been reduced by the learning acquired in the first session of interaction with the game, that is, the players learned that after reaching the target and receiving the visual and sound stimuli, they should perform the required movement. Thus, although the player performed the movements more slowly, he was able to initiate the execution of the movements earlier.

After participants 3 and 14 took the medication, the movements were performed faster, that is, movement time was decreased, but RT increased, which consequently resulted in increased reaction time. Movement time may have been reduced due to the medication’s effectiveness in reducing motor symptoms. The reaction time may have increased due to the lack of learning that could have been acquired in the first session; that is, the participants did not understand that the visual stimulus indicated the exact moment to start the execution of the movement. Thus, although the motor symptoms were reduced, the players did not learn enough, making them start the execution of the movements later.

Finally, after participants 4, 8, and 13 took the medication, their RT increased, and they performed the movements more slowly, that is, they increased the movement time. It can be suggested that the medication does not reduce the motor symptoms of these individuals; therefore, a readjustment of the medication dosage should be performed. Moreover, these individuals probably did not acquire learning in relation to the game.

Medication is the key treatment for the motor symptoms of PD, but the pharmacological response inevitably diminishes over time [46]. Following these results, it is possible to identify the individuals who possibly need medication adjustments, as well as those who require additional training with the serious game to better understand its features and rules. These findings may provide a more personalized patient follow-up by health care professionals and assist them in assessing individuals and monitoring the disease.

In phase 1, the median RT of the EG-OFF was lower than that of both the EG-ON and the CG. The EG-ON also showed lower RTs compared to the healthy controls. However, no statistically significant differences were found between the EG-OFF and EG-ON groups, whereas both EGs differed significantly from the CG. One possible explanation for this pattern is that participants in the EG had already been exposed to the RehaBEElitation serious game, which may have provided a degree of familiarity with the task demands. This prior exposure may have facilitated faster motor planning and reduced hesitancy during the initial phases of interaction, even among participants in the OFF-medication condition. In contrast, healthy controls, despite not presenting motor symptoms, were inexperienced with the task and may have required additional time to internalize the stimuli and response mapping, resulting in comparatively longer reaction times. It is worth noting, however, that the CG exhibited a narrower or flatter boxplot distribution in phase 1, indicating lower variability and greater consistency in performance compared to the EGs. A similar trend was observed in phase 3, in which the EG-ON demonstrated lower RTs than the healthy controls, although without reaching statistical significance. Together, these findings suggest that task familiarity may have contributed to the observed differences in RT performance.

Furthermore, the information indicated by Figures 5-7 showed that, in general, the RT and AV scores for the CG participants were better than those for the EG in the ON state, which in turn were better than those for the EG in the OFF state, as already expected. In addition, EG participants in the OFF state of medication and CG participants who presented the worst results for RT also presented the worst results for AV, which reveals that the measures used for the assessment of slowness were quite consistent.

According to Figures5  6, phase 4 is the most suitable for assessing slowness using the RehaBEElitation serious game. Figure 5 shows that, in this phase, the differences between the groups were better detected, since the boxplots overlapped less, and only in phase 4 was it possible to estimate the intentional AV of the participants (Figure 6). Movements performed around the other axes, such as flexion-extension around the X axis and adduction-abduction around the Z axis, were not used to score points but to steer the bee within the scenario. Because these actions served navigation, players could deliberately modulate them (eg, moving more slowly or with reduced amplitude) to fine-tune the bee’s positioning. Consequently, AV derived from these axes would not reflect performance intent and was not analyzed. In contrast, when a movement is executed to score points, it is performed with intentional speed and amplitude, which are suitable for AV estimation. For this, AV analyses were restricted to the gyroscope signals recorded in phase 4.

A further point to discuss is that phase 4 required the execution of the forearm supination and pronation movement, a more complex movement than the others and one that is not often performed during daily activities. This may have encouraged players to focus on the execution of the movement, which may have influenced a better estimation of the variables used in the study. These findings agree with those reported by Summa et al [47].

Effect size estimates provide important information about the practical relevance of the differences observed between groups. Although all phases of the RT analysis showed statistically significant differences, the associated effect sizes were small for phases 2 and 3, small-to-moderate for phase 1, and moderate for phase 4, suggesting that group membership explains only a limited part of the variability in RTs. This indicates that, although the groups are different, other factors such as individual variability, familiarity with the task, or motor strategy may play a substantial role in RT performance. In contrast, the AV results revealed a large effect size (η^2^=0.404), indicating that the differences between groups in AV are not only statistically significant but also of great practical magnitude. This pattern also confirms that AV is a more sensitive marker of group-specific motor behavior than RT and, therefore, may serve as a stronger discriminator of motor performance differences in this context.

The grouped visualization shown in Figure 7 highlights an overall pattern of motor performance between groups but does not capture individual nuances or intrasubject variations. These differences may be related to the heterogeneity of symptoms among people with PD, variability in response to medication, level of familiarity with the game, motivation, rigidity, and different difficulties present in tasks at each phase of the game. Thus, although the overall trend indicates greater slowness in the Parkinson group, future studies should consider more detailed analyses at the individual level, as well as increasing the number of trials per participant, to reduce motor compensations or momentary fluctuations and obtain even more robust measures.

Correlation analysis revealed distinct patterns between RT and AV variables and the motor items of the MDS-UPDRS. While RT showed very weak to weak correlations with items that reflect slowness on the clinical scale, AV showed strong and consistent correlations, indicating, once again, greater sensitivity of this metric to the typical motor impairment of PD. In this study, slowness was estimated from the sum of reaction time and movement time (RT variable), although reaction time is not part of the clinical assessment of this symptom. This conceptual difference may have contributed to the weaker correlations observed between RT and MDS-UPDRS scores. Additionally, RT seems to reflect broader aspects of interaction with the task, such as attention, familiarity, and individual strategies, which may explain the lower magnitude of the associations.

### Strengths and Limitations

An important contribution of this study is the definition of a specific event within the game that functions as a behavioral marker, allowing the estimation of a variable indicative of slowness (RT) during task execution. Although not a biological marker, this event provides a structured and reproducible reference point from which RT can be calculated. As already discussed, this variable, despite not showing a strong correlation with the MDS-UPDRS, captures a central component of motor slowness, as it reflects both the preparation and initiation of movement, processes known to be impaired in PD. Without this event, it would not be possible to quantify slowness with the RT variable in the simple way that was carried out. There is a total of 23 predefined markers in the game, which were detailed in a study describing the architecture and organization of our system [17]. Thus, the game’s internal markers play a key role in providing objective anchors within the interaction, allowing the derivation of meaningful measures of motor performance.

Study limitations include the lack of prior training for the CG and the single-day assessment for the EG in both ON and OFF medication states. While this scheduling avoided collection bias, the cumulative duration of tasks may have impacted performance during the ON-state trials for some participants. Additionally, the PD group used their most affected limb, while the CG used their dominant limb. Although this introduces variability, it preserves ecological validity, as bradykinesia is characteristically asymmetric. Since our goal was to characterize game interaction under realistic clinical conditions rather than compare absolute performance, we consider the impact of this difference on our findings to be limited.

### Future Directions

In this study, the generalization of the results is influenced by the sample characteristics. Although the use of a serious game that presents a well-established motor task favors broader applicability, the results should be interpreted, considering that participants presented mild to moderate PD (Hoehn and Yahr I-III) and that all interactions with the system were conducted in a controlled environment. Therefore, extrapolation to individuals with advanced disease, other clinical contexts, or unsupervised home use should be done with caution. Future studies with larger and more heterogeneous samples are needed to confirm the external validity of these results.

In comparison to other methodologies, using serious games is an appealing option for measuring Parkinson motor symptoms. This game can be applied frequently and easily in clinics and hospitals, which demonstrates excellent accessibility and is cost-effective. In addition, the methods applied can isolate voluntary activity from involuntary activity. Severe tremor interferes with the classification of bradykinesia.

This study advances the current literature by proposing a novel and objective method to quantify slowness in PD using inertial sensor data acquired during interaction with a serious game. While previous studies have explored serious games for PD assessment and rehabilitation, most relied on subjective clinical scales, questionnaires, or conventional physical tests to evaluate motor symptoms. In contrast, our approach directly analyzes sensor-derived metrics obtained during gameplay, enabling the extraction of quantitative measures of RT and AV without interrupting the user experience. To the best of our knowledge, this is the first study to evaluate slowness in PD by processing inertial sensor data collected during interaction with a serious game. This contribution strengthens the role of serious games as potential digital biomarkers and highlights their value as engaging tools for continuous and sensitive motor assessment. From a real-world perspective, this approach may facilitate remote monitoring in home environments, complement clinical evaluations, and support personalized follow-up of motor symptom progression, thereby expanding access to objective and technology-driven care for individuals with PD.

### Conclusions

The findings of this study suggest that the RehaBEElitation serious game is a promising tool for objectively assessing motor slowness in individuals with PD. Interaction-derived variables, particularly AV, showed strong correlations with MDS-UPDRS motor scores, capturing motor impairment sensitively. The game also detected differences between ON and OFF medication states, reinforcing the ability of the serious game to detect changes associated with treatment. Overall, these results indicate that the RehaBEElitation serious game represents a viable and potentially valuable approach for the objective monitoring of slowness and for supporting strategies for tracking the progression and therapeutic management of PD.

## Supplementary material

10.2196/79463 Multimedia Appendix 1 Phases of the game. (A) Phase 1: pollinating the flowers. The image shows that the player has collected pollen and had his or her hand closed, as indicated by the yellow grains below the bee. (B) Phase 2: feeding the larvae. When the player opens his or her hand to deliver food to the larvae and feed them, the light arcs above the larvae disappear. (C) Phase 3: collecting the nectar. As the player approaches the water drops, it is necessary to make a pinching gesture to collect it. (D) Phase 4: drying the nectar. When the honeycombs containing nectar are dried (after pronation and supination movement execution), the light reflex disappears.

10.2196/79463 Multimedia Appendix 2 Wearable human-machine interface based on a glove in 2 different perspectives. A compact casing positioned on the back of the hand houses the electronic circuitry and related components responsible for transmitting motion data to the game.

10.2196/79463 Multimedia Appendix 3 Schematic of the experimental scenario (lateral view). The participant was positioned comfortably in a chair to facilitate the best possible interaction with the game. The researcher intervened when necessary, fostering a more human-centered experimental session.

10.2196/79463 Multimedia Appendix 4 Response time definition. It represents the total response duration, encompassing both the reaction time (from stimulus onset to movement initiation) and the movement time (from movement initiation to task completion). Lower values indicate faster performance.

10.2196/79463 Multimedia Appendix 5 Sensor axis orientation. Movements on the other axes were used only to steer the bee in the scenario (not to score points) and could be intentionally slowed down or minimized; therefore, angular velocity analyses were restricted to phase 4.

10.2196/79463 Multimedia Appendix 6 Overview of the singular spectrum analysis decomposition pipeline, covering 4 steps: embedding, decomposition, grouping, and reconstruction.

10.2196/79463 Multimedia Appendix 7 Decomposition of a gyroscope signal from an experimental group participant. The top signal represents the original time series, containing both components of voluntary movement (from gameplay) and involuntary movement (tremor). The middle signal isolates the voluntary movement, that is, the intentional control of the bee. The bottom signal isolates the disease-related tremor, that is, only the oscillatory or periodic components of the original time series.

## Abbreviations

AV: angular velocity
CG: control group
EG: experimental group
HMI: human-machine interface
IMU: inertial measurement unit
MDS-UPDRS: Movement Disorder Society-Unified Parkinson’s Disease Rating Scale
PD: Parkinson disease
RT: response time
SSA: singular spectrum analysis
SSD: singular spectrum decomposition
SVD: singular value decomposition
UFU: Federal University of Uberlandia
