# Supplementary figures and images for: Quantifying Slowness in Parkinson Disease Using a Serious Game: Cross-Sectional Study
Source: JMIR Serious Games. 2026 Feb 25;14:e79463. doi: 10.2196/79463 (PMC12935423; doi:10.2196/79463)

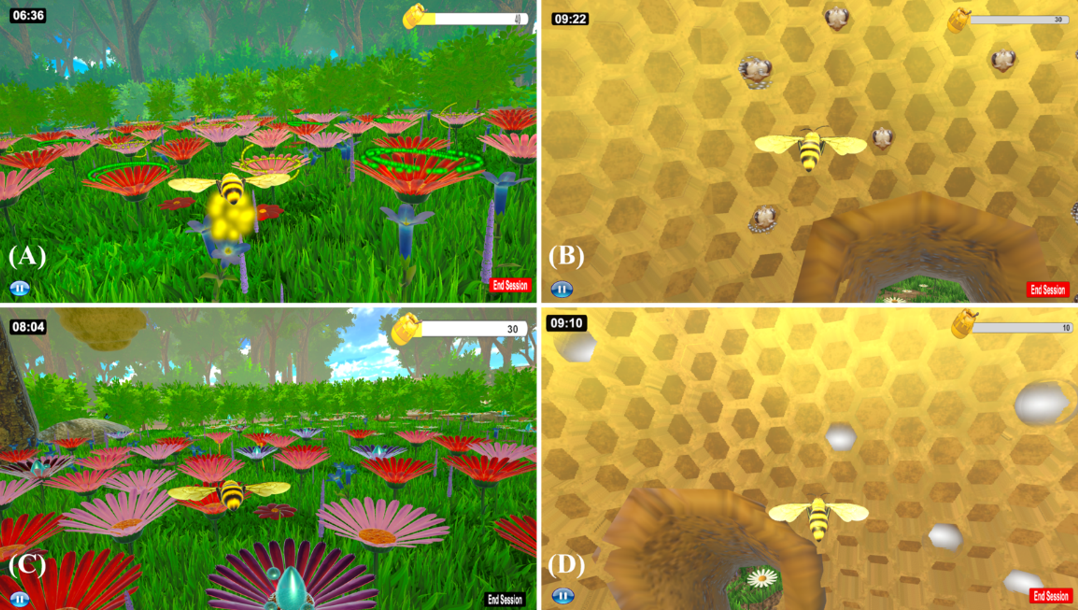

Supplement: Multimedia Appendix 1 [file games-v14-e79463-s001.png]

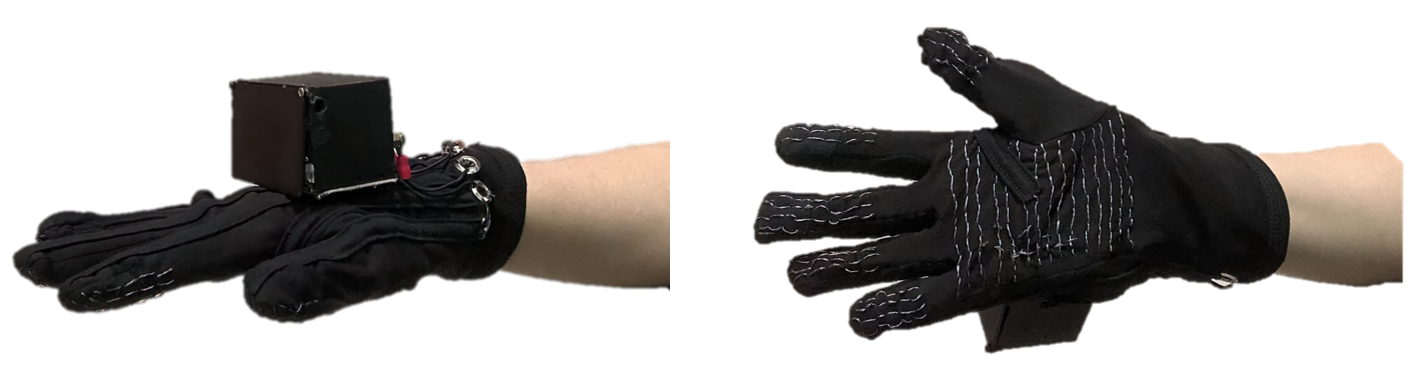

Supplement: Multimedia Appendix 2 [file games-v14-e79463-s002.png]

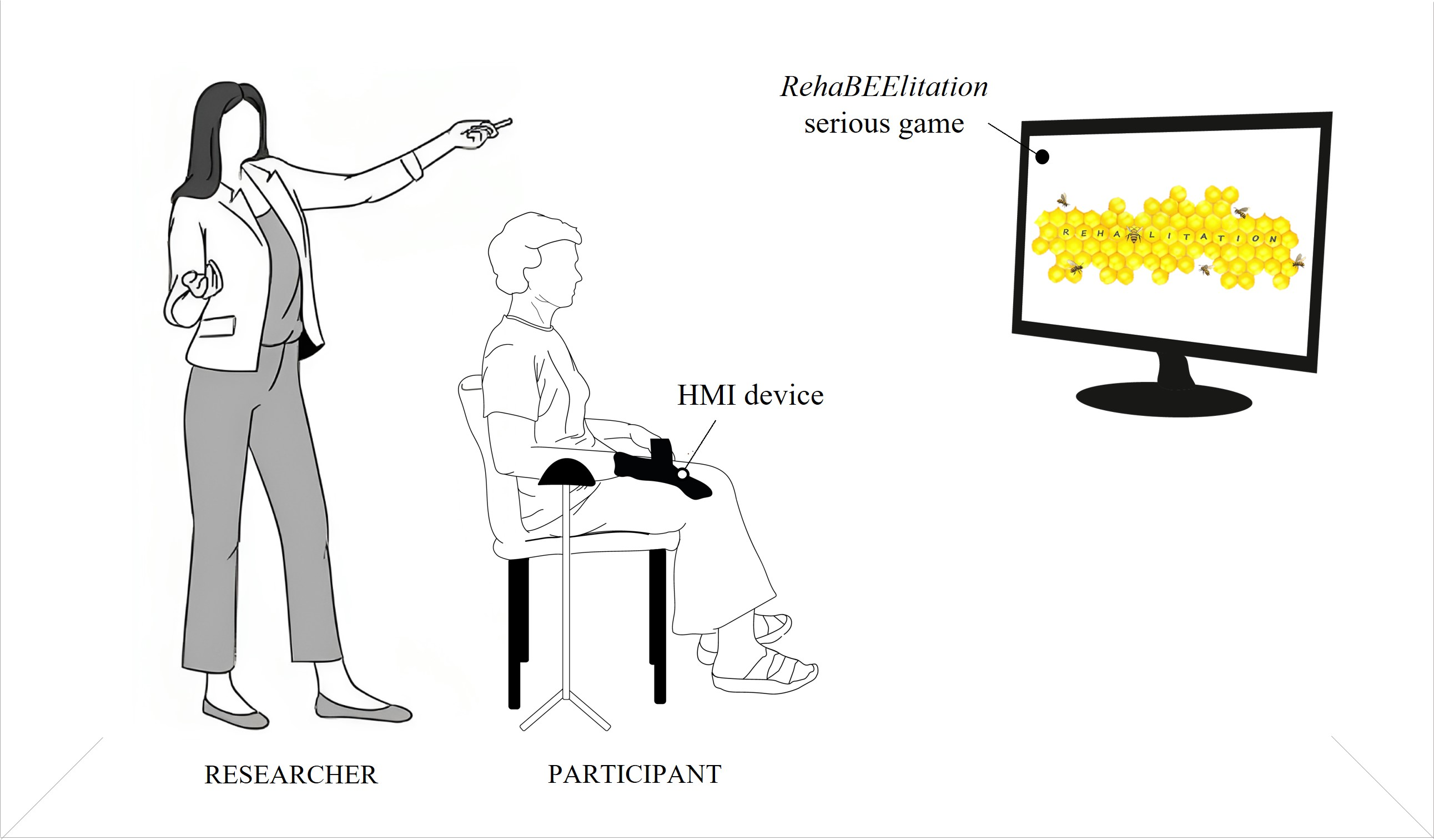

Supplement: Multimedia Appendix 3 [file games-v14-e79463-s003.png]

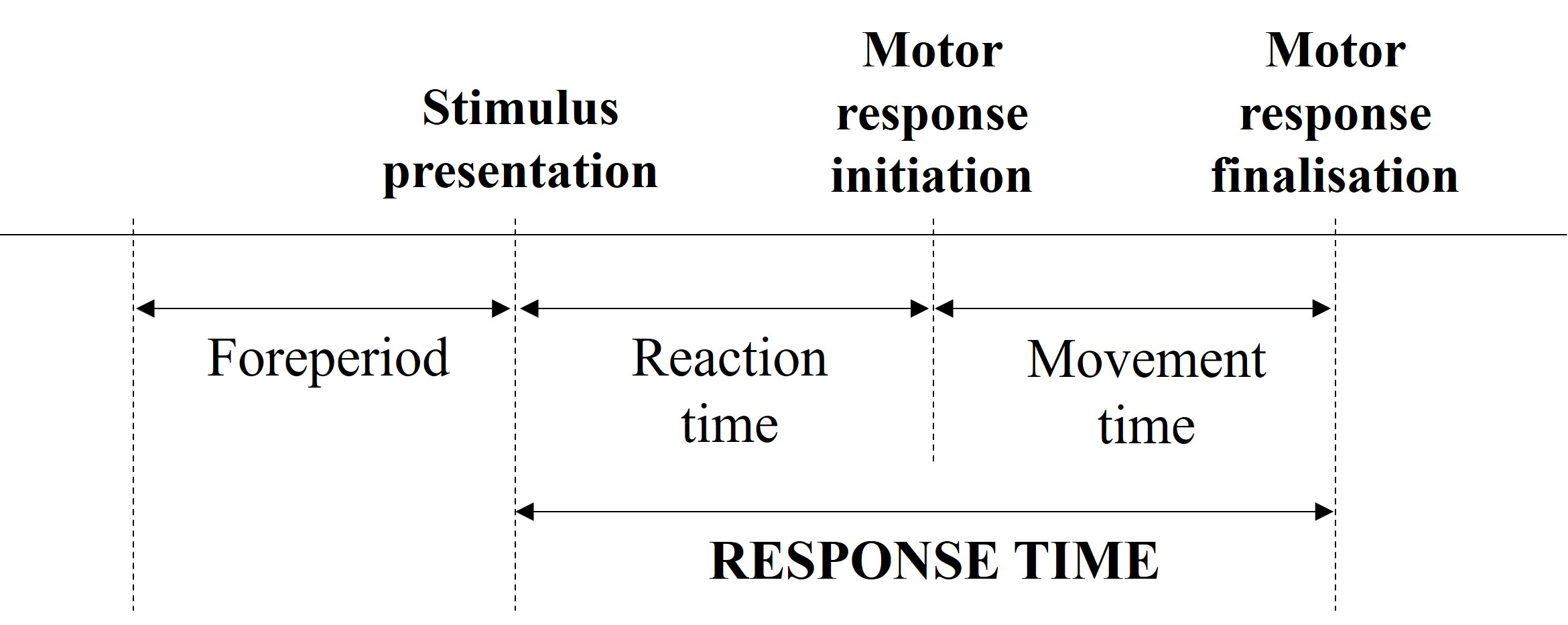

Supplement: Multimedia Appendix 4 [file games-v14-e79463-s004.png]

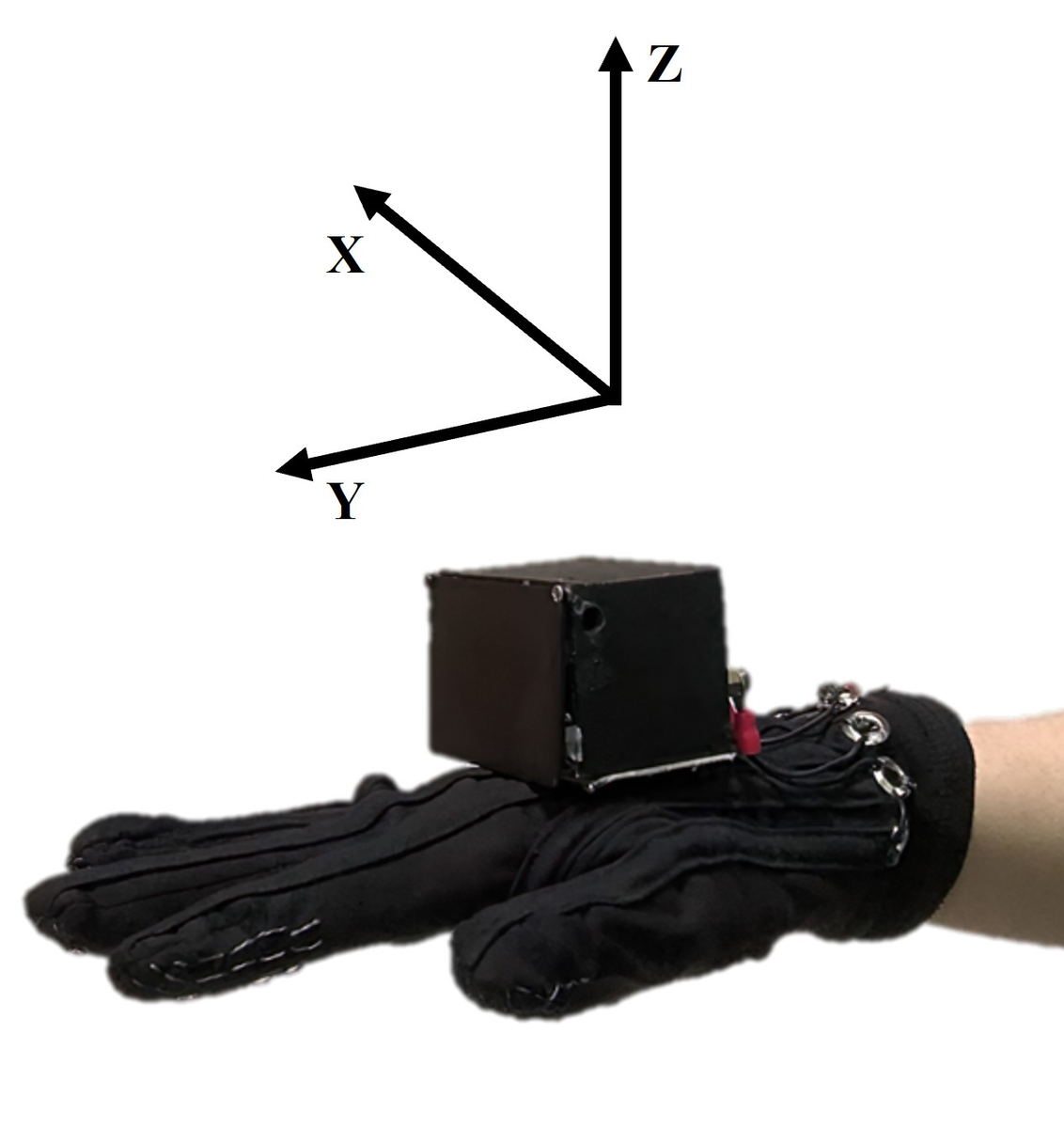

Supplement: Multimedia Appendix 5 [file games-v14-e79463-s005.png]

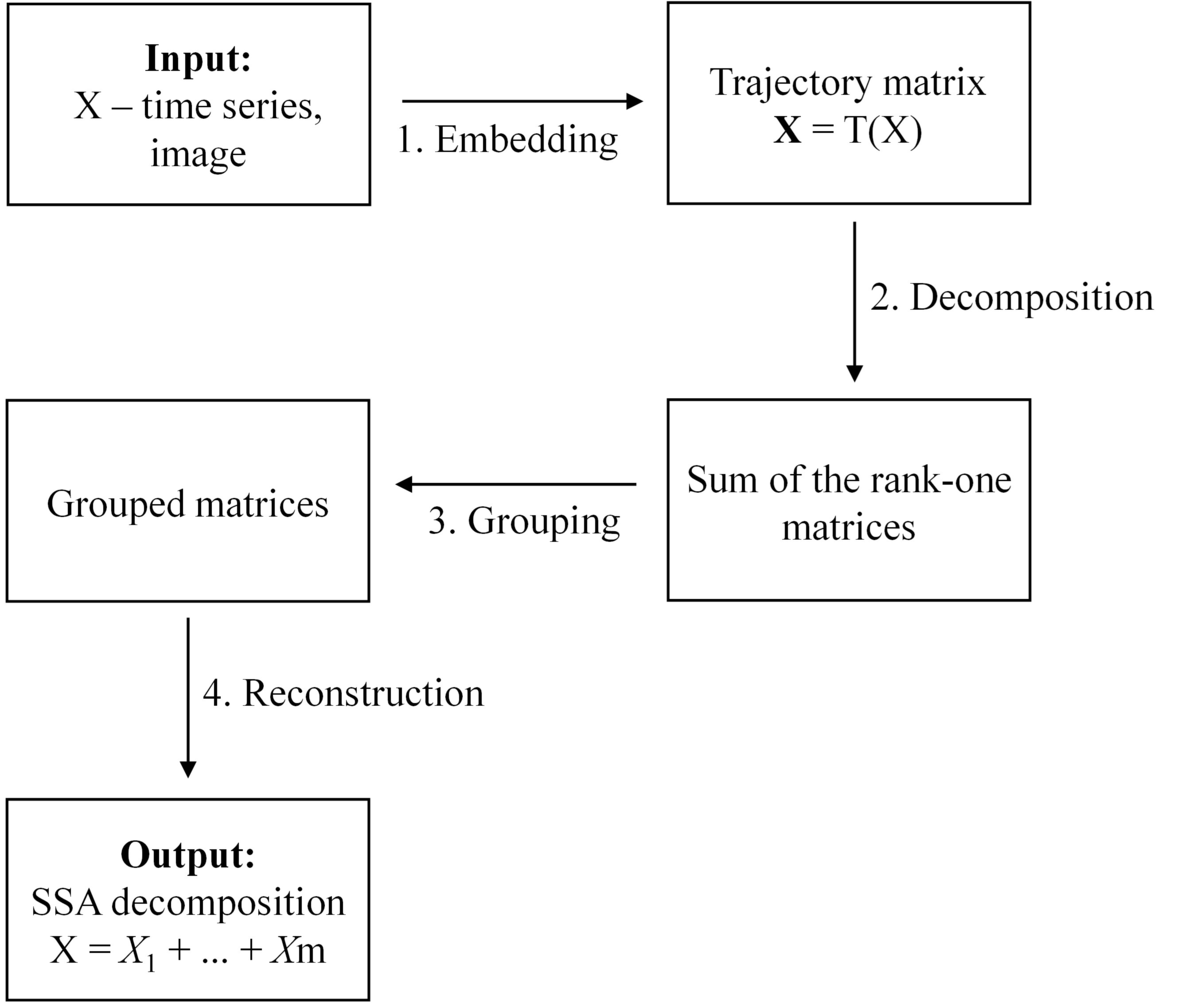

Supplement: Multimedia Appendix 6 [file games-v14-e79463-s006.png]

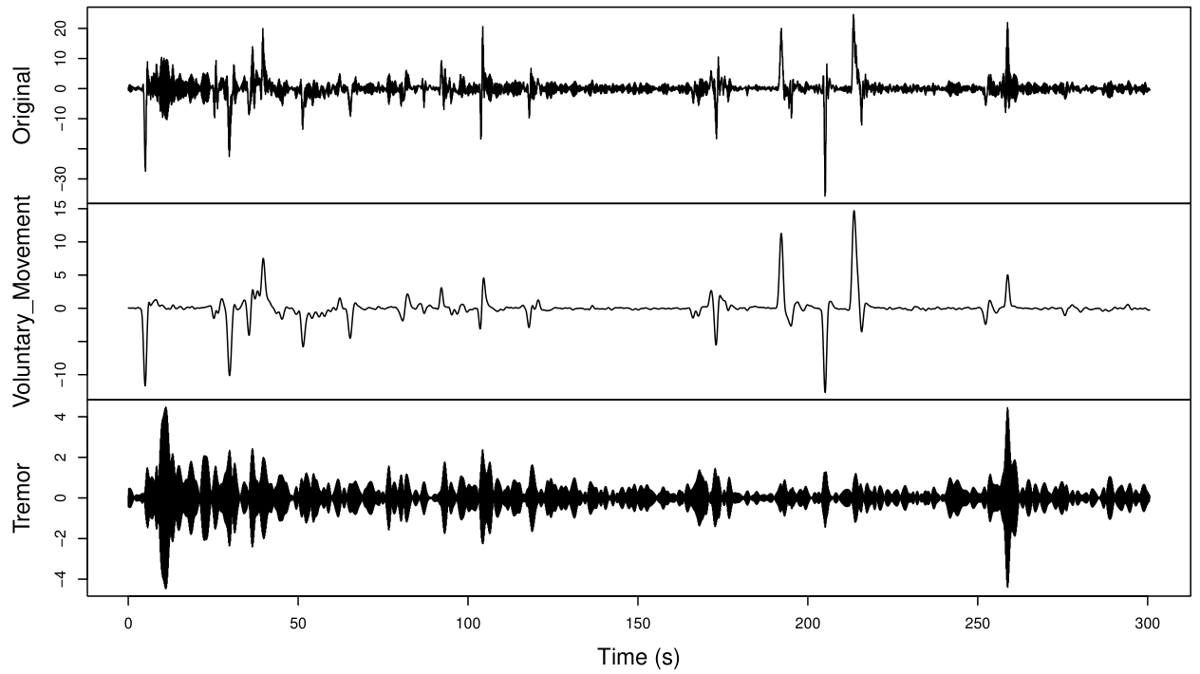

Supplement: Multimedia Appendix 7 [file games-v14-e79463-s007.png]
